# Systemic immune-inflammation index and fibrinogen-to-albumin ratio as predictors of coronary collateral circulation in chronic total occlusion patients

**DOI:** 10.3389/fcvm.2026.1777321

**Published:** 2026-03-31

**Authors:** Xi Wu, Haobo Huang, Kai Hu, Lei Wang, Xia Wu, He Huang, Mingxing Wu

**Affiliations:** Department of Cardiology, Xiangtan Central Hospital (the Affiliated Hospital of Hunan University), Xiangtan, Hunan, China

**Keywords:** chronic total occlusion, coronary collateral circulation, systemic immune-inflammation index, fibrinogen-to-albumin ratio, biomarkers

## Abstract

**Background:**

Coronary collateral circulation (CCC) plays a vital compensatory role in maintaining myocardial perfusion in patients with chronic total occlusion (CTO) of the coronary arteries. Systemic inflammation and nutritional status are known to influence arteriogenesis; however, the roles of the systemic immune-inflammation index (SII) and the fibrinogen-to-albumin ratio (FAR) in CCC formation remain underexplored.

**Objective:**

This study aimed to evaluate the association between SII and FAR levels and the development of CCC in patients with CTO.

**Methods:**

A retrospective analysis was conducted on 469 patients with coronary chronic total occlusion who underwent elective coronary angiography. Patients were stratified into poorly-developed and well-developed CCC groups based on the Rentrop collateral grading system. Baseline clinical characteristics, laboratory biomarkers, and angiographic findings were compared between groups. Univariate and multivariate logistic regression analyses were performed to identify independent predictors of poorly-developed CCC. The predictive value of SII and FAR, both individually and in combination, was evaluated using receiver operating characteristic (ROC) curve analysis.

**Results:**

Patients with poorly-developed CCC exhibited significantly higher levels of SII and FAR compared to those with well-developed CCC. In multivariate logistic regression, both SII [odds ratio [OR] = 3.121, 95% confidence interval [CI]: 1.827–5.537, *P* < 0.001] and FAR (OR = 2.118, 95% CI: 1.248–3.227, *P* = 0.001) emerged as independent predictors of poor collateral formation. The combined use of SII and FAR yielded improved discriminatory performance, with an area under the ROC curve (AUC) of 0.73.Correlation analysis revealed that SII (*r* = –0.377, *P* < 0.001) and FAR (*r* = –0.815, *P* < 0.001) were negatively correlated with Rentrop collateral grades. Notably, FAR showed consistent statistical significance across all grades, suggesting that elevated inflammation and nutritional imbalance are strongly associated with impaired coronary collateral development.

**Conclusion:**

Elevated SII and FAR are independently associated with poorly-developed CCC in patients with CTO. These biomarkers may serve as simple, cost-effective tools for clinical risk stratification and decision-making. Further prospective studies are needed to validate their prognostic utility and therapeutic implications in CCC modulation.

## Introduction

Chronic total occlusion (CTO) of the coronary arteries is characterized by a complete arterial blockage with Thrombolysis In Myocardial Infarction (TIMI) grade 0 antegrade flow, persisting for an estimated duration of ≥3 months (1). The pathophysiology involves rupture of atherosclerotic plaques, subsequent thrombus formation, and failure of spontaneous recanalization, ultimately resulting in sustained vascular occlusion (2). Owing to its chronic evolution and intricate lesion morphology, CTO presents significant challenges in percutaneous intervention, with lower revascularization success rates and poorer prognostic outcomes compared to non-CTO lesions (3).

Coronary collateral circulation (CCC) denotes a network of pre-formed vascular conduits that interconnect branches within or between coronary arteries (4). These vessels typically exhibit minimal flow under physiological conditions and hold limited functional significance. However, in the context of severe coronary stenosis or occlusion, they undergo adaptive enlargement via arteriogenesis, reaching diameters between 100 and 800 *μ*m. This remodeling facilitates the restoration of downstream myocardial perfusion (5). In CTO cases, effective CCC helps sustain myocardial viability, limits infarct extent, supports preserved contractile function, and contributes to enhanced clinical prognosis and patient survival (6, 7). Additionally, a well-developed collateral network lowers the incidence of complications such as papillary muscle rupture, ventricular septal defect, and left ventricular aneurysm, while providing retrograde access for guidewire navigation during PCI, thereby improving procedural outcomes and alleviating ischemic symptoms (8).

Numerous factors influence the extent and efficacy of CCC, including patient age, presence of diabetes mellitus, dyslipidemia, hypertension, duration and severity of stenosis, diastolic pressure, endothelial function, and oxidative stress status (9). Inflammation is also a pivotal contributor not only to the initiation and progression of coronary atherosclerosis but also to the modulation of collateral vessel growth (10). Existing evidence suggests that elevated inflammatory biomarkers—such as the neutrophil-to-lymphocyte ratio (NLR), platelet-to-lymphocyte ratio (PLR), and C-reactive protein—are associated with impaired collateral development in patients with stable coronary artery disease (11).

The systemic immune-inflammation index (SII), derived from neutrophil, platelet, and lymphocyte counts, represents a novel integrated inflammatory indicator. It has been linked to adverse outcomes in various malignancies (12), and more recently, its prognostic relevance has been demonstrated in patients with acute decompensated chronic heart failure and in predicting major adverse cardiovascular events following percutaneous coronary intervention (PCI) (13). Furthermore, elevated SII has shown associations with the no-reflow phenomenon post-PCI in myocardial infarction cases (13). Nonetheless, its potential impact on CCC development in CTO patients remains insufficiently investigated.

Fibrinogen (FIB) and albumin (ALB), both hepatic synthesis products, reflect distinct biological processes. FIB plays a critical role in hemostasis by interacting with platelet glycoprotein IIb/IIIa receptors, facilitating platelet aggregation (14). This interaction promotes the formation of microthrombi, contributing to microvascular obstruction and disease progression (15). Conversely, ALB is vital for maintaining oncotic pressure and ensuring metabolic homeostasis (16). Hypoalbuminemia has been correlated with accelerated cardiovascular disease, adverse prognosis, and increased mortality (17). The fibrinogen-to-albumin ratio (FAR), combining proinflammatory and nutritional indicators, has emerged as a meaningful marker in inflammatory bowel disease, metastatic cancer, and thromboembolic disorders (18). However, its relevance to coronary collateral development in the context of CTO has yet to be comprehensively clarified.

Accordingly, this study aims to explore the relationship between SII and FAR levels and the formation of CCC in patients with CTO. By examining their predictive capacity, the findings may offer novel insights into risk stratification and optimization of clinical strategies for managing these patients.

## Materials and methods

### Study participants

This retrospective, single-center observational study was carried out in the Department of Cardiology at Xiangtan Central Hospital from June 2018 to December 2024. A total of 469 patients were consecutively enrolled based on predefined inclusion and exclusion criteria. All patients had been hospitalized and underwent elective coronary angiography (CAG), which identified at least one CTO in a major coronary artery. The definition of CTO adhered to the 2019 European CTO Club guidelines (19), specifying a complete coronary occlusion with TIMI grade 0 antegrade flow, absence of angiographic thrombus, presence of a well-defined proximal fibrous cap, and an estimated occlusion duration of ≥3 months. The duration of occlusion was estimated by reviewing clinical documentation or through follow-up communication, and was based on indicators such as the initial occurrence of worsening angina, prior myocardial infarction involving the affected vascular territory, or comparative data from previous angiographic or imaging examinations.

**Figure 1:**
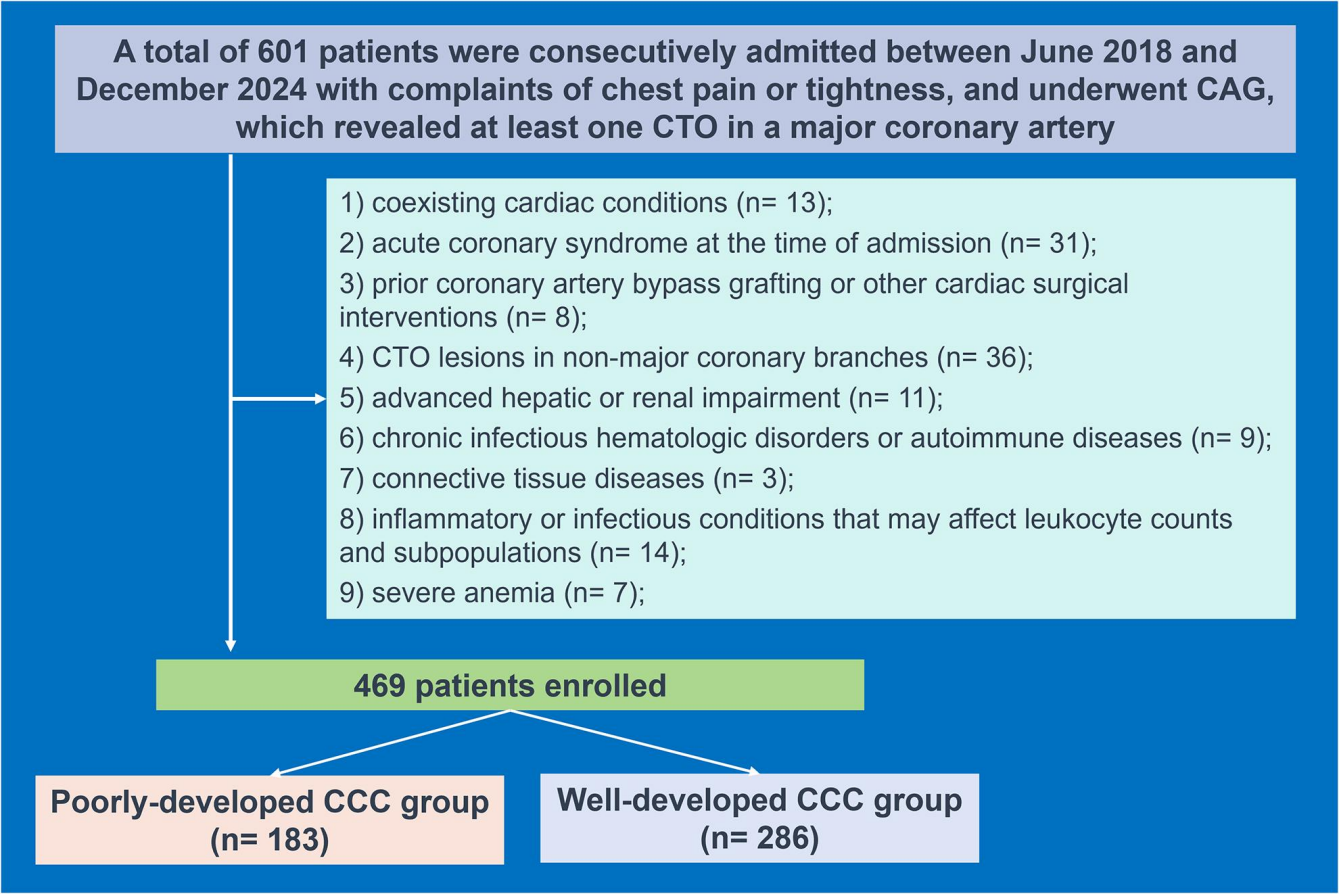
Study flowchart. CCC, coronary collateral circulation; CTO, chronic total occlusion; CAG, coronary angiography.

Participants were eligible if comprehensive clinical data were available, including routine hematological assessments, lipid profiles, liver and renal function parameters, and echocardiographic findings. Exclusion criteria encompassed coexisting cardiac conditions such as valvular heart disease, congenital heart anomalies, viral myocarditis, or cardiomyopathies; acute coronary syndrome at the time of admission; prior coronary artery bypass grafting (CABG) or other cardiac surgical interventions; CTO lesions in non-major coronary branches; advanced hepatic or renal impairment; hematologic disorders; chronic infectious or autoimmune diseases; connective tissue diseases; inflammatory or infectious conditions that may affect leukocyte counts and subpopulations; severe anemia; acute myocardial infarction necessitating emergency PCI during its acute phase and recent major trauma or surgery within the past month (Figure 1).

The study protocol received ethical approval from the Ethics Committee of Xiangtan Central Hospital (Approval No. X2019523) and was conducted in accordance with the principles outlined in the Declaration of Helsinki (2013 revision). Written informed consent was obtained from all participants. In cases where written documentation was not feasible, verbal consent was acquired and formally recorded in accordance with institutional guidelines.

### Data collection

#### Baseline clinical characteristics

Clinical data for all enrolled patients were obtained from the hospital's electronic medical record system. Baseline characteristics included age, sex, body mass index (BMI), admission blood pressure, and medical histories such as hypertension, diabetes mellitus, and stroke. Information on lifestyle habits (e.g., smoking status) and previous medication use was also collected.

#### Laboratory measurements

Fasting venous blood samples were drawn on the morning of the second day post-admission to assess hematological and biochemical profiles. These included complete blood count, fasting glucose, liver and renal function markers, lipid panel, N-terminal pro-B-type natriuretic peptide (NT-ProBNP), and cardiac biomarkers. Inflammatory and composite indices were calculated as follows:

SII = neutrophil count × platelet count/lymphocyte count
FAR = FIB/ALB
NLR = neutrophil/lymphocyte
PLR = platelet/lymphocyte
Neutrophil-to-Albumin Ratio (NAR) = neutrophil/albumin

#### Coronary angiographic assessment

All participants underwent elective CAG, performed via either radial or femoral arterial access. Angiographic images were independently evaluated by two experienced interventional cardiologists. In the event of discordant evaluations, a third cardiologist provided adjudication. Angiographic assessments included identification of the diseased artery, number of affected vessels, lesion location, stenosis severity, presence and degree of CCC, and Rentrop collateral classification (20).

The extent of coronary atherosclerosis was quantified using the Gensini scoring system (21), which assigns weights based on lesion severity and anatomical location. Scores were calculated by multiplying stenosis grade (ranging from 1 for 1%–25% to 32 for total occlusion) by a segment-specific weighting factor, with the cumulative score reflecting overall coronary disease burden. Collateral filling was graded using the Rentrop classification: Grade 0: No collateral filling; Grade 1: Filling of side branches only; Grade 2: Partial filling of the epicardial artery via collaterals; Grade 3: Complete epicardial filling through collateral vessels.

A vessel was considered occluded if it demonstrated 100% luminal obstruction with TIMI grade 0 flow. In patients with multiple occluded arteries, the one with visible collateral supply was designated the target vessel. If more than one vessel met these criteria, the artery with the highest Rentrop grade was selected. When Rentrop grades were equal, the more proximal vessel was prioritized.

#### Study group stratification

Based on collateral flow grading, patients were categorized into two groups: Poorly-developed CCC group: Rentrop grades 0–1; Well-developed CCC group: Rentrop grades 2–3. Comparisons of demographic, clinical, and laboratory parameters were made between the two groups.

### Statistical analysis

Statistical analysis was performed using SPSS version 26.0 (IBM Corp., Armonk, NY, USA). Normality of continuous variables was evaluated using the Shapiro–Wilk test. Normally distributed data were presented as mean ± standard deviation (SD) and analyzed using independent-sample t tests. Non-normally distributed variables were expressed as median (interquartile range, IQR) and compared using the Mann–Whitney *U* test. Categorical variables were reported as frequencies and percentages and assessed using the chi-square test. Associations between continuous variables and CCC status (coded as 0 = poorly-developed, 1 = well-developed) were examined using point-biserial correlation for normally distributed data and rank-biserial correlation for non-normal data. Univariate logistic regression identified potential predictors of poorly-developed CCC. Variables with *P* < 0.05 in univariate analysis were entered into multivariate logistic regression to determine independent predictors. To assess and control for potential multicollinearity among the variables, we calculated the variance inflation factor (VIF) for each predictor variable. Variables with a VIF greater than 10 were carefully examined for multicollinearity, and adjustments were made to ensure the robustness of the regression model. This approach helped in identifying and mitigating the influence of multicollinearity in the statistical analysis. The combined SII and FAR were evaluated using a combined ROC analysis. To construct the combined model, SII and FAR were entered into a logistic regression model to generate a composite score, which was then used for receiver operating characteristic (ROC) curve analysis. This combined model was assessed for its ability to predict poorly-developed CCC. The performance of this combined model was compared to that of the individual SII and FAR markers using DeLong test for area under the curve (AUC) comparisons. ROC curves were constructed using MedCalc version 20.11 (MedCalc Software Ltd, Ostend, Belgium). A two-tailed *P* value < 0.05 was considered statistically significant.

## Results

### Baseline clinical characteristics and laboratory biomarkers

Among the 469 patients with CTO included in this analysis, 183 individuals (39.0%) were classified into the poorly-developed CCC group, while 286 individuals (61.0%) constituted the well-developed CCC group. No statistically significant differences were observed between the two groups in terms of demographic characteristics (age, sex), smoking status, medical histories (hypertension, diabetes mellitus, stroke), or baseline clinical parameters including systolic blood pressure, diastolic blood pressure, heart rate, and BMI (all *P* > 0.05) (Table 1).

**Table 1:** Baseline clinical characteristics and laboratory biomarkers between poorly-developed CCC group and well-developed CCC group.

Variable	ALL (*n* = 469)	Poorly-developed CCC group (*n* = 183)	Well-developed CCC group (*n* = 286)	*P* value
Age, years	68.50 ± 3.78	68.59 ± 3.60	68.44 ± 3.90	0.677
Male, *n*%	270 (57.6%)	103 (56.3%)	167 (58.4%)	0.722
Smoking history, *n* %	224 (47.8%)	95 (51.9%)	129 (45.1%)	0.178
Hypertension, *n* %	235 (50.1%)	98 (53.6%)	137 (47.9%)	0.271
Diabetes mellitus, *n* %	125 (26.7%)	54 (29.5%)	71 (24.8%)	0.311
Prior history of stroke, *n* %	99 (21.1%)	33 (18.0%)	66 (23.1%)	0.234
SBP, mmHg	134.68 ± 18.88	135.49 ± 17.81	134.15 ± 19.51	0.456
DBP, mmHg	81.98 ± 11.98	82.32 ± 10.67	81.77 ± 12.75	0.629
Heart rate, bpm	75.59 ± 13.68	76.67 ± 13.13	74.90 ± 13.99	0.173
Body mass index, kg/m^2^	25.07 ± 2.67	24.99 ± 2.26	25.12 ± 2.90	0.611
Laboratory biomarkers				
White blood cell, ×10^9^/L	6.87 ± 1.53	7.09 ± 1.53	6.73 ± 1.52	0.011
Neutrophil count, ×10^9^/L	4.38 ± 1.27	4.95 ± 1.30	4.02 ± 1.11	<0.001
Lymphocyte count, ×10^9^/L	1.83 ± 0.63	1.62 ± 0.55	1.97 ± 0.63	<0.001
Monocyte count, ×10^9^/L	0.51 (0.45,0.57)	0.50 (0.45, 0.56)	0.52 (0.45, 0.58)	0.203
Eosinophil count, ×10^9^/L	0.09 (0.06,0.12)	0.09 (0.06, 0.13)	0.09 (0.06, 0.12)	0.346
Basophil count, ×10^9^/L	0.03 (0.02,0.04)	0.03 (0.02, 0.04)	0.03 (0.02, 0.04)	0.681
Red blood cell count, ×10^12^/L	4.68 (4.49, 4.88)	4.71 (4.50, 4.89)	4.67 (4.46, 4.88)	0.285
Hemoglobin concentration, g/L	143.97 (138.48, 149.63)	144.16 (138.81, 149.37)	143.71 (138.43, 149.82)	0.532
Platelet count, ×10^9^/L	229.38 ± 48.44	240.43 ± 48.03	222.31 ± 47.37	<0.001
Fasting blood glucose, mmol/L	5.94 (5.44, 6.47)	5.86 (5.54, 6.33)	6.01 (5.39, 6.62)	0.145
Glycated hemoglobin, %	6.10 (5.63, 6.56)	6.06 (5.69, 6.45)	6.12 (5.59, 6.68)	0.193
Fibrinogen, g/L	3.01 (2.83, 3.19)	3.19 (3.02, 3.36)	2.90 (2.71, 3.07)	<0.001
hs-CRP, mg/L	3.81 ± 2.01	5.17 ± 2.18	2.93 ± 1.29	<0.001
Blood urea nitrogen, mmol/L	6.06 (5.38, 6.80)	6.13 (5.41, 6.82)	6.02 (5.36, 6.76)	0.249
Uric acid, μmol/L	346.14 (312.19, 393.79)	342.97 (295.69, 394.50)	346.95 (316.36, 391.09)	0.069
Creatinine, μmol/L	77.25 (70.63, 84.09)	77.04 (70.32, 87.50)	77.41 (71.17, 82.79)	0.299
eGFR, mL/min/1.73 m^2^	91.79 (86.37, 97.10)	92.25 (87.19, 97.54)	91.49 (85.81, 96.62)	0.070
ALT, U/L	21.60 (16.91, 26.58)	22.29 (17.51, 26.89)	21.33 (16.11, 25.91)	0.066
AST, U/L	19.03 (16.28, 22.16)	19.03 (16.82, 21.62)	19.04 (15.47, 22.77)	0.370
Albumin, g/L	41.28 (40.09, 42.58)	41.03 (39.86, 42.61)	41.47 (40.18, 42.56)	0.079
Globulin, g/L	26.14 (25.07, 27.14)	26.16 (24.85, 27.05)	26.13 (25.19, 27.17)	0.266
Total bilirubin, μmol/L	12.14 (10.61, 13.91)	12.33 (10.72, 14.13)	11.98 (10.58, 13.72)	0.145
Direct bilirubin, μmol/L	3.34 (2.89, 3.87)	3.37 (2.83, 3.85)	3.34 (2.91, 3.87)	0.735
Indirect bilirubin, μmol/L	8.61 (7.49, 9.78)	8.37 (7.28, 9.77)	8.81 (7.60, 9.77)	0.186
Triglycerides, mmol/L	1.58 (1.33, 1.86)	1.60 (1.34, 1.93)	1.56 (1.32, 1.84)	0.162
Total cholesterol, mmol/L	3.77 (3.52, 4.06)	3.84 (3.46, 4.18)	3.74 (3.55, 4.02)	0.103
HDL-C, mmol/L	0.855 (0.795, 0.908)	0.903 (0.842, 0.959)	0.822 (0.763, 0.883)	<0.001
LDL-C, mmol/L	2.483 (2.207, 2.775)	2.472 (2.227, 2.818)	2.488 (2.201, 2.759)	0.401
Apolipoprotein B, mg/dL	84.555 (76.679, 91.374)	84.908 (76.291, 93.095)	84.189 (76.791, 90.700)	0.380
Lipoprotein(a), mg/L	193.840 (97.842, 288.977)	202.178 (100.181, 310.371)	191.267 (97.876, 276.421)	0.307
cTNI, ng/mL	0.010 (0.005, 0.016)	0.010 (0.005, 0.016)	0.010 (0.005, 0.016)	0.734
NT-ProBNP, pg/mL	482.99 ± 715.69	508.04 ± 762.34	466.97 ± 683.70	0.545
SII, 10^9^/L	515.3 (359.6, 682.7)	670.7 (360.2, 986.8)	473.4 (358.6, 583.8)	<0.001
FAR	0.075 (0.069, 0.081)	0.082 (0.079, 0.085)	0.071 (0.067, 0.074)	<0.001
NLR	2.58 ± 1.19	3.15 ± 1.52	2.22 ± 0.71	<0.001
PLR	157.33 ± 19.63	154.45 ± 16.42	159.17 ± 21.23	0.011
LVEF, %	50.61 ± 5.10	49.92 ± 4.74	51.06 ± 5.27	0.018

Continuous variables were expressed as mean ± SD, or median (interquartile range). Categorical variables were expressed as number (percentage).

CCC, coronary collateral circulation; SBP, systolic blood pressure; DBP, diastolic blood pressure; hs-CRP, high-sensitivity C-reactive protein; eGFR, estimated glomerular filtration rate; ALT, alanine aminotransferase; AST, aspartate aminotransferase; HDL-C, high-density lipoprotein cholesterol; LDL-C, low-density lipoprotein cholesterol; cTNI, cardiac troponin I; NT-ProBNP, N-terminal pro-B-type natriuretic peptide; SII, systemic immune-inflammation index; FAR, fibrinogen-to-albumin ratio; NLR, neutrophil-to-lymphocyte ratio; LVEF, left ventricular ejection eraction; DAPT, dual antiplatelet therapy; ACEI, angiotensin-converting enzyme inhibitor; ARB, angiotensin-receptor blocker; PLR, platelet-to-lymphocyte ratio.

In terms of laboratory parameters, patients in the poorly-developed CCC group exhibited significantly higher levels of white blood cell count (7.09 ± 1.53 vs. 6.73 ± 1.52 × 10⁹/L, *P* = 0.011), neutrophil count (4.95 ± 1.30 vs. 4.02 ± 1.11 × 10⁹/L, *P* < 0.001), platelet count (240.43 ± 48.03 vs. 222.31 ± 47.37 × 10⁹/L, *P* < 0.001), FIB (3.19 vs. 2.90 g/L, *P* < 0.001), and high-sensitivity C-reactive protein (5.17 ± 2.18 vs. 2.93 ± 1.29 mg/L, *P* < 0.001). Additionally, inflammatory indices including SII [670.7 (360.2, 986.8) vs. 473.4 (358.6, 583.8), *P* < 0.001], FAR [0.082 (0.079, 0.085) vs. 0.071 (0.067, 0.074), *P* < 0.001], and NLR (3.15 ± 1.52 vs. 2.22 ± 0.71, *P* < 0.001) were markedly elevated in the poorly-developed CCC group, accompanied by a significantly lower lymphocyte count (1.62 ± 0.55 vs. 1.97 ± 0.63 × 10⁹/L, *P* < 0.001). Conversely, PLR (159.17 ± 21.23 vs. 154.45 ± 16.42, *P* = 0.011) and high-density lipoprotein cholesterol (HDL-C) levels (0.822 vs. 0.903 mmol/L, *P* < 0.001) were significantly higher in the well-developed CCC group. Left ventricular ejection fraction (LVEF) was slightly reduced in the poorly-developed group compared to the well-developed group (49.92 ± 4.74% vs. 51.06 ± 5.27%, *P* = 0.018) (Table 1).

### Angiographic findings and Pre-admission medications

No significant differences were observed between the two groups in terms of lesion distribution in the left anterior descending coronary artery (LAD) (49.2% vs. 40.9%, *P* = 0.096) or left circumflex coronary artery (LCX) (26.8% vs. 28.3%, *P* = 0.795). However, a significantly higher proportion of right coronary artery (RCA)-CTO lesions was found in the well-developed CCC group compared to the poorly-developed group (55.9% vs. 44.8%, *P* = 0.023). The incidence of multi-vessel CTOs did not differ significantly (12.0% vs. 14.3%, *P* = 0.563) (Table 2).

**Table 2:** Angiographic findings and Pre-admission medication.

Variable	ALL (*n* = 469)	Poorly-developed CCC group (*n* = 183)	Well-developed CCC group (*n* = 286)	*P* value
Angiographic findings				
CTO lesion location, *n* %				
LAD	207 (44.1%)	90 (49.2%)	117 (40.9%)	0.096
LCX	130 (27.7%)	49 (26.8%)	81 (28.3%)	0.795
RCA	242 (51.6%)	82 (44.8%)	160 (55.9%)	0.023
Multi-vessel CTO lesions, *n* %	63 (13.4%)	22 (12.0%)	41 (14.3%)	0.563
Gensini score	62.00 (47.00, 78.00)	42.00 (34.00, 51.00)	75.00 (65.00, 86.00)	<0.001
Pre-admission medication				
DAPT, *n* %	418 (89.1%)	168 (91.8%)	250 (87.4%)	0.181
Statins, *n* %	347 (74.0%)	139 (76.0%)	208 (72.7%)	0.503
ACEI or ARB, *n* %	220 (46.9%)	78 (42.6%)	142 (49.7%)	0.163
Beta-blockers, *n* %	237 (50.5%)	89 (48.6%)	148 (51.7%)	0.573
Aldosterone antagonists, *n* %	41 (8.7%)	18 (9.8%)	23 (8.0%)	0.614
Nitrates, *n* %	205 (43.7%)	88 (48.1%)	117 (40.9%)	0.151
Calcium channel blockers, *n* %	88 (18.8%)	38 (20.8%)	50 (17.5%)	0.443

Continuous variables were expressed as median (interquartile range). Categorical variables were expressed as number (percentage).

CCC, coronary collateral circulation; LAD, left anterior descending coronary artery; LCX, left circumflex coronary artery; RCA, right coronary artery; CTO, chronic total occlusion; ACEI, angiotensin-converting enzyme inhibitor; ARB, angiotensin-receptor blocker;.

Patients in the well-developed CCC group exhibited higher Gensini scores than those in the poorly-developed group [75.00 (65.00, 86.00) vs. 42.00 (34.00, 51.00), *P* < 0.001], indicating more severe coronary artery disease burden (Table 2).

There were no significant intergroup differences in the use of medications prior to admission, including dual antiplatelet therapy (91.8% vs. 87.4%, *P* = 0.181), statins (76.0% vs. 72.7%, *P* = 0.503), angiotensin-converting enzyme inhibitor (ACEI) or angiotensin-receptor blocker (ARB) (42.6% vs. 49.7%, *P* = 0.163), beta-blockers (48.6% vs. 51.7%, *P* = 0.573), aldosterone antagonists (9.8% vs. 8.0%, *P* = 0.614), nitrates (48.1% vs. 40.9%, *P* = 0.151), or calcium channel blockers (20.8% vs. 17.5%, *P* = 0.443) (Table 2).

### Univariate and multivariate logistic regression analysis

Logistic regression analysis was performed to identify independent predictors of poorly-developed CCC. In univariate analysis, multiple variables were initially assessed. After adjusting for potential confounders in multivariate models (Table 3), both SII [odds ratio (OR) = 3.121, 95% confidence interval (CI): 1.827–5.537, *P* < 0.001] and FAR (OR = 2.118; 95% CI: 1.248–3.227; *P* = 0.001) emerged as independent predictors of impaired collateral development in CTO patients.

**Table 3:** Univariate and multivariate regression analyses for the influential factors of poorly-developed CCC formation in CTO patients.

	Univariate Analysis			Multivariate Analysis			
Variables	OR	95% CI	*P* value	OR	95% CI	*P* value	VIF
White blood cell, ×10^9^/L	0.991	0.964–1.027	0.933				1.13
Neutrophil count, ×10^9^/L	1.071	0.583–1.982	0.825				1.31
lymphocyte count, ×10^9^/L	0.933	0.535–1.637	0.817				1.18
Platelet count, ×10^9^/L	1.205	0.681–2.128	0.531				1.25
Fibrinogen, g/L	0.827	0.412–1.675	0.527				1.12
hs-CRP, mg/L	1.737	0.932–3.136	0.075				1.27
HDL-C, mmol/L	0.773	0.332–1.628	0.537				1.05
SII, 10^9^/L	4.275	2.721–6.467	<0.001	3.121	1.827–5.537	<0.001	7.48
FAR	1.626	1.221–2.275	0.002	2.118	1.248–3.227	0.001	4.52
NLR	1.073	0.831–1.337	0.437				7.57
PLR	1.074	0.947–1.271	0.147				8.20
LVEF, %	0.937	0.901–1.036	0.337				1.37
CTO lesion location at RCA	4.026	2.247–7.745	<0.001	2.471	0.974–4.327	0.073	3.22
Gensini score	1.014	1.004–1.047	0.025	1.000	0.943–1.044	0.936	2.54

CCC, coronary collateral circulation; CTO, chronic total occlusion; OR, odds ratio; CI, confidence interval; RCA, right coronary artery; SII, systemic immune-inflammation index; FAR, fibrinogen-to-albumin ratio; NLR, neutrophil-to-lymphocyte ratio; LVEF, left ventricular ejection eraction; PLR, platelet-to-lymphocyte ratio; VIF, variance inflation factor;.

### Correlation between SII, FAR, and CCC development

**Figure 2:**
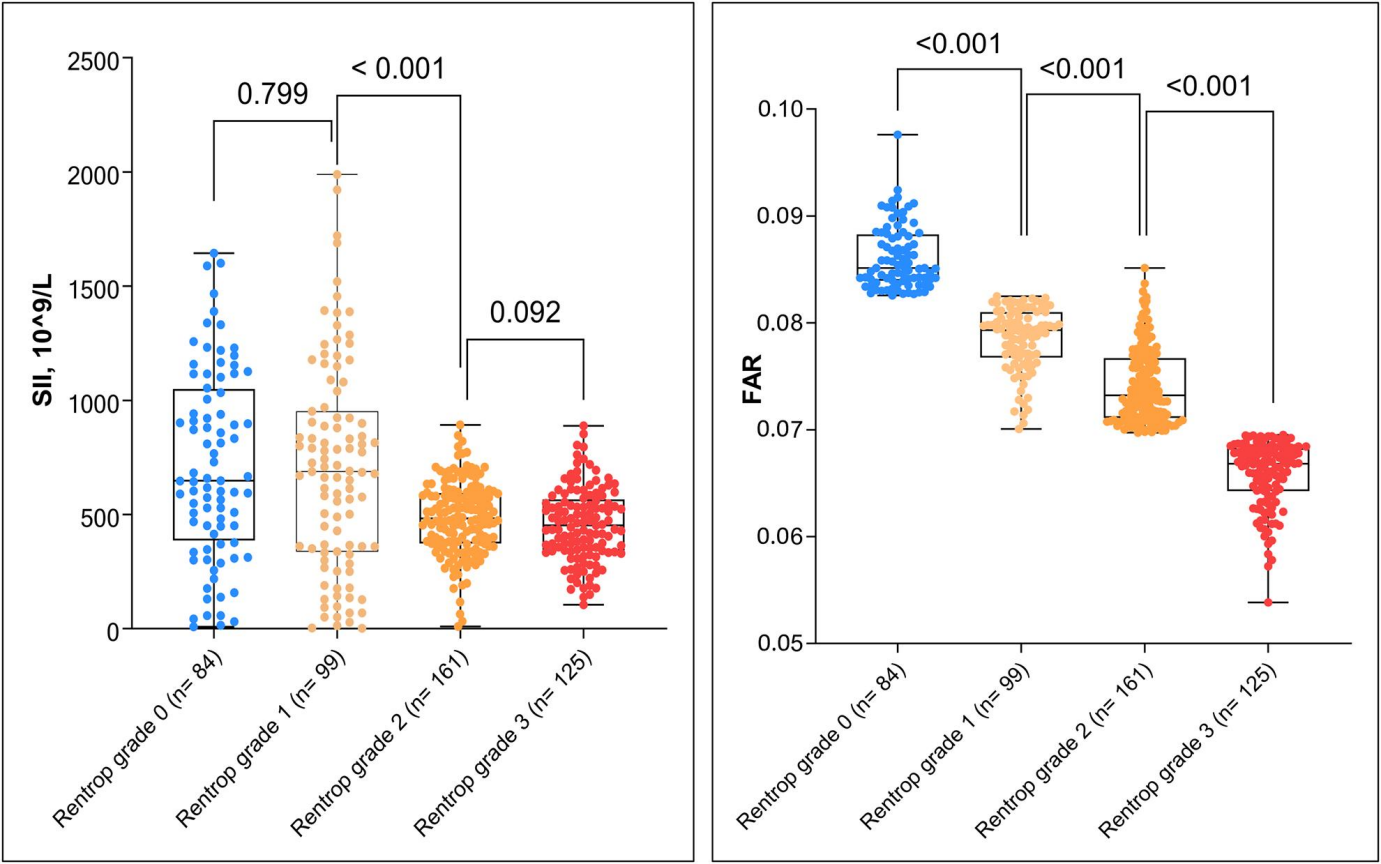
Comparisons of SII and FAR levels across Rentrop grades. **(A)** Distribution of SII across Rentrop grade 0 to 3 groups. **(B)** Distribution of FAR across Rentrop grades. Data are presented as box plots with overlaid individual data points. Statistically significant differences (*P* < 0.001) are indicated above the brackets. SII, systemic immune-inflammation index; FAR, fibrinogen-to-albumin ratio.

To further elucidate the relationship between inflammatory markers and collateral circulation, SII and FAR levels were compared across different Rentrop grades (Table 4, Figure 2). A declining trend in both markers was observed with increasing Rentrop grade. Median SII values were: Grade 0: 647.86 (405.28, 1039.08); Grade 1: 688.20 (344.55, 938.05); Grade 2: 482.53 (374.67, 592.81); Grade 3: 452.48 (341.28, 560.45). The only significant difference was found between grades 1 and 2 (*P* < 0.001), whereas differences between grade 0 vs. 1 (*P* = 0.799) and grade 2 vs. 3 (*P* = 0.092) were not statistically significant. FAR levels showed more consistent significance: Grade 0: 0.085 (0.084, 0.088); Grade 1: 0.079 (0.077, 0.081); Grade 2: 0.073 (0.071, 0.077); Grade 3: 0.067 (0.064, 0.068).

**Table 4:** Comparison of SII and FAR across Rentrop grades.

Variable	Rentrop grade 0 (*n* = 84)	Rentrop grade 1 (*n* = 99)	Rentrop grade 2 (*n* = 161)	Rentrop grade 3 (*n* = 125)	*P* value
SII, 10^9^/L	647.86 (405.28, 1,039.08)	688.20 (344.55, 938.05)	482.53 (374.67, 592.81)	452.48 (341.28, 560.45)	0.799, <0.001, 0.092
FAR	0.085 (0.084, 0.088)	0.079 (0.077, 0.081)	0.073 (0.071, 0.077)	0.067 (0.064, 0.068)	<0.001

Continuous variables were expressed as median (interquartile range).

*P* values were calculated using independent samples *t*-test for SII and Mann–Whitney U test for FAR across sequential Rentrop grade comparisons (0 vs. 1, 1 vs. 2, 2 vs. 3).

SII: Among the pairwise comparisons between Rentrop grades, only the difference between Grade 1 and Grade 2 was statistically significant (*P* < 0.001). Grade 0 vs. 1 (*P* = 0.799) and Grade 2 vs. 3 (*P* = 0.092) were not statistically significant.FAR: All sequential group comparisons are statistically significant (*P* < 0.001).

SII, systemic immune-inflammation index; FAR, fibrinogen-to-albumin ratio;.

Each adjacent grade comparison was statistically significant (*P* < 0.001). Correlation analysis confirmed that both SII and FAR were negatively associated with Rentrop grade. Specifically, SII exhibited a moderate inverse correlation (r = –0.377, *P* < 0.001), while FAR demonstrated a strong negative correlation (r = –0.815, *P* < 0.001), indicating that elevated inflammation and nutritional imbalance are associated with poorer collateral vessel development.

### ROC curve analysis

**Figure 3:**
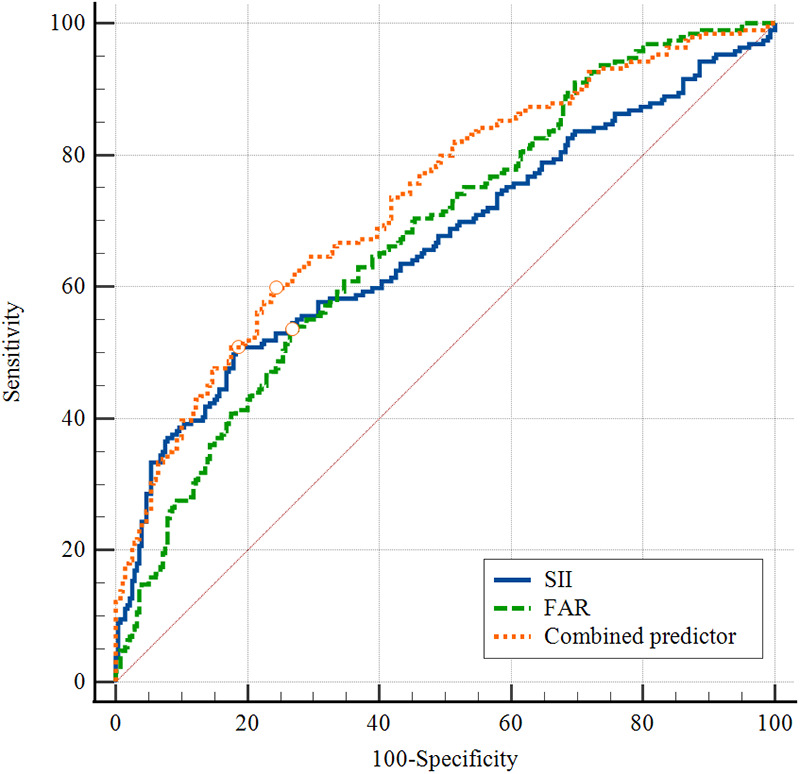
ROC curves for SII, FAR, and the combined predictor in predicting poorly-developed CCC formation in CTO patients. ROC, receiver operating characteristic; CCC, coronary collateral circulation; CTO, chronic total occlusion; SII, systemic immune-inflammation index; FAR, fibrinogen-to-albumin ratio.

The diagnostic value of SII, FAR, and their combination for predicting poorly-developed CCC was assessed using ROC curves (Figure 3, Table 5). SII: AUC = 0.67 (95% CI: 0.61–0.72); cutoff = 631.51; sensitivity = 50.8%; specificity = 81.4%. FAR: AUC = 0.68 (95% CI: 0.63–0.73); cutoff = 0.077; sensitivity = 53.4%; specificity = 73.2%. Combined SII + FAR: AUC = 0.73 (95% CI: 0.68–0.78); sensitivity = 59.8%; specificity = 75.7%. The comparison of AUCs for SII, FAR, and the combined model was performed using the DeLong test. The results showed that the combined SII + FAR model had a significantly higher AUC compared to SII (*P* = 0.02) and FAR (*P* = 0.03), indicating that the combination of these two biomarkers enhances diagnostic accuracy for identifying patients at risk of insufficient collateral vessel formation.

**Table 5:** ROC curve analysis of individual and combined indicators in predicting poorly-developed CCC formation in CTO patients.

Variable	AUC (95% CI)	Best cutoff	Sensitivity	Specificity	*P* value		
					SII	FAR	Combined predictor
SII	0.67 (0.61–0.72)	631.51	50.8%	81.4%	-	0.743	0.001
FAR	0.68 (0.63–0.73)	0.077	53.4%	73.2%	0.743	-	0.004
Combined predictor	0.73 (0.68–0.78)	-	59.8%	75.7%	0.001	0.004	-

CI, confidence interval; ROC, receiver operator characteristic; AUC, area under the curve; SII, systemic immune-inflammation index; FAR, fibrinogen-to-albumin ratio.

## Discussion

In this retrospective analysis of 469 patients diagnosed with CTO, we explored the relationship between SII, FAR, and the extent of CCC. Our findings revealed that patients with poorly-developed CCC exhibited significantly elevated levels of both SII and FAR when compared to those with well-developed collateral networks. These observations imply that heightened systemic inflammation and nutritional imbalance may impair the formation of coronary collateral vessels. Multivariate logistic regression analysis confirmed that both SII and FAR independently predicted poorly-developed CCC, even after adjusting for confounding clinical and biochemical variables. Notably, the combined model incorporating both markers demonstrated enhanced predictive capacity, with an AUC of 0.73, indicative of moderate diagnostic performance. To our knowledge, this study is one of the first to systematically assess the combined prognostic utility of SII and FAR in the context of CCC development among CTO patients. These results emphasize the integral role of inflammatory activity and metabolic status in regulating arteriogenesis, offering novel biomarkers for risk stratification and potentially guiding personalized management approaches in CTO care (Central Illustration).

**Central Illustration:**
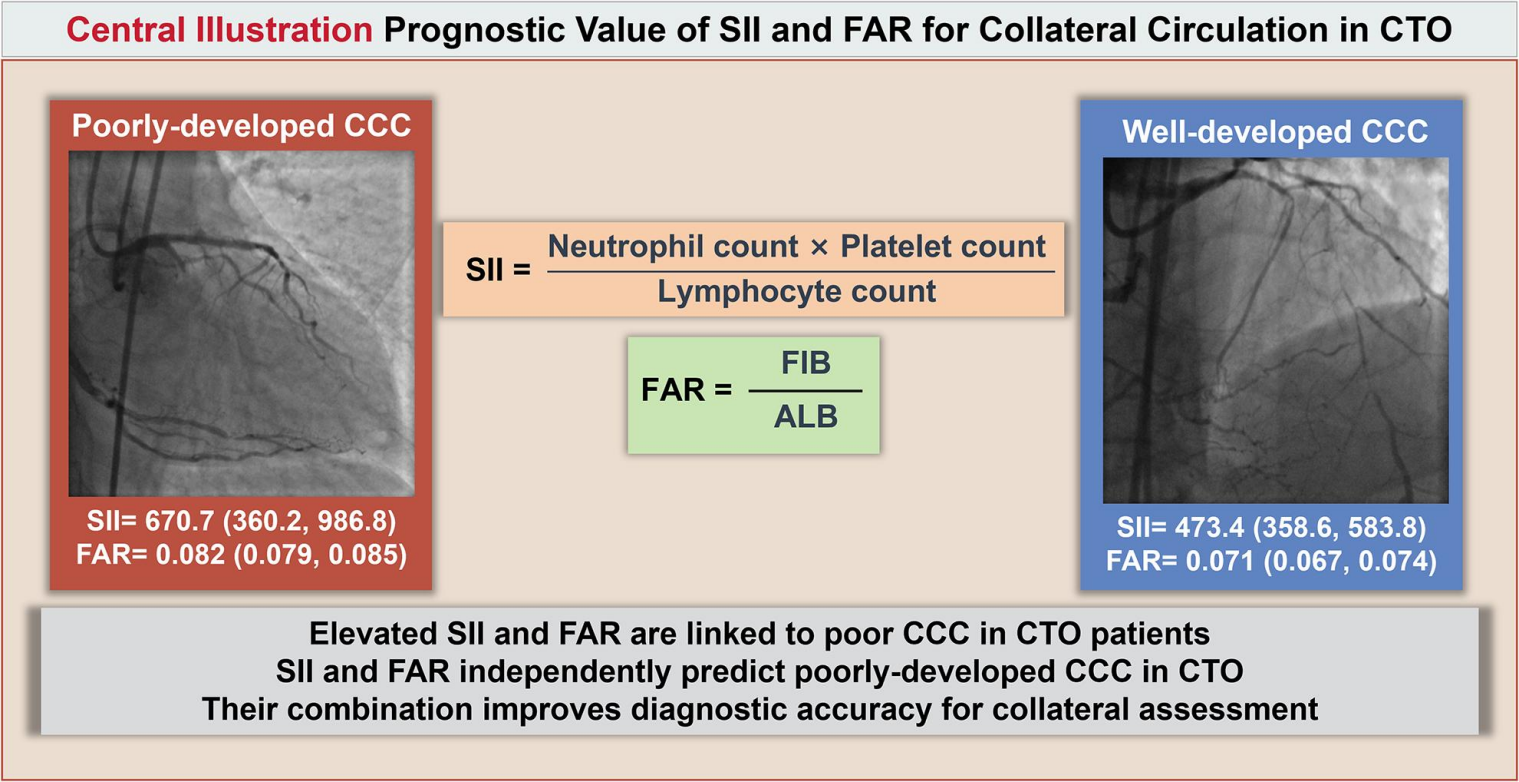
Prognostic role of SII and FAR for CCC in CTO patients. This schematic summarizes the association between SII and FAR with the development of CCC in patients with CTO. Patients with poorly-developed CCC exhibited significantly higher levels of SII and FAR compared to those with well-developed CCC. SII and FAR were independent predictors of inadequate collateral formation, and their combination improved diagnostic accuracy (AUC = 0.73). These markers reflect systemic inflammation and nutritional status and may serve as practical tools for clinical risk stratification and therapeutic decision-making. SII, systemic immune-inflammation index; FAR, fibrinogen-to-albumin ratio; FIB, fibrinogen; ALB, albumin; CCC, coronary collateral circulation; CTO, chronic total occlusion.

CTOs are histopathologically composed of smooth muscle cells, extracellular matrix components, neovascularized tissue, calcium deposits, and lipid accumulation (22). The occlusion site typically demonstrates columnar fibrotic and calcific structures at its proximal and distal ends, encasing central regions with thrombotic materials and lipid-rich cores (23). A growing body of experimental and pathological studies supports that the genesis of CTO lesions is initiated by endothelial dysfunction and lipid infiltration, triggered by immune cell activation and proinflammatory cytokines. These inflammatory cascades promote smooth muscle cell proliferation and macrophage infiltration within the intima, driving maladaptive remodeling and progressive luminal obstruction (24). These mechanistic insights align with our clinical findings, further supporting the concept that inflammation plays a pervasive and continuous role throughout the entire course of CTO lesion formation and evolution.

### The role of SII in coronary collateral circulation development

This study identified a significant inverse association between the SII and the extent of CCC in patients with CTO. SII, a composite marker that incorporates neutrophil, lymphocyte, and platelet counts, has been extensively evaluated in oncology settings (12, 25), and its clinical relevance in cardiovascular diseases is now gaining increasing attention. In our study population, elevated SII levels were independently associated with poorly-developed CCC, as confirmed through multivariate logistic regression analysis.

Among the range of inflammatory indicators assessed—such as white blood cell count, NLR, and PLR—only SII consistently demonstrated predictive value for inadequate collateral development, emphasizing its broader scope in capturing inflammatory status. Our findings are partially aligned with those of Kalkan et al., who reported significant intergroup differences in white blood cells, NLR, and C-reactive protein (CRP) between well- and poorly-developed CCC groups (26). However, in contrast to their results, we observed that NLR did not retain statistical significance in multivariate analysis. Similarly, Açar et al. identified both PLR and NLR as predictive markers in CTO patients with stable angina (11). These inconsistencies may be attributed to differences in sample size, potential multicollinearity among markers, or variations in study populations. Given that SII integrates three distinct inflammatory components, it may offer a more comprehensive and reliable measure of systemic inflammatory burden compared to NLR or PLR individually.

Biologically, systemic inflammation interferes with arteriogenesis through several mechanisms including endothelial dysfunction, leukocyte adhesion and infiltration, and pro-inflammatory cytokine release, all of which hinder the maturation of functional collateral networks. Neutrophils, for instance, are rapidly recruited to sites of vascular injury and secrete reactive oxygen species and proteases that compromise endothelial integrity (27). They also influence angiogenesis through granulocyte colony-stimulating factor (G-CSF)-mediated modulation of vascular endothelial growth factor (VEGF) production and progenitor cell mobilization (28). Lymphocytes support arteriogenesis by coordinating the recruitment and activation of monocytes and macrophages; T-cell deficiency has been shown to impede collateral vessel formation in ischemic conditions (29). Platelets contribute dual roles by releasing both pro-angiogenic factors such as VEGF, platelet-derived growth factor (PDGF), and basic fibroblast growth factor (bFGF), and anti-angiogenic agents like angiostatin, which inhibit nitric oxide synthesis and may suppress collateral development (30).

Furthermore, chronic inflammation can elevate circulating platelet counts and mean platelet volume through cytokine-driven megakaryocyte activation (31). By integrating neutrophilic, lymphocytic, and thrombocytic responses, SII effectively captures the multifaceted immune-inflammatory milieu that contributes to impaired arteriogenesis. Our correlation analysis reinforces this perspective, revealing a significant negative association between SII and Rentrop grade, where higher SII values corresponded to diminished collateral vessel formation.

In conclusion, SII emerges as a practical and comprehensive biomarker reflecting the dynamic interplay between inflammation, immune response, and vascular remodeling. Compared to conventional markers such as NLR or PLR, SII appears to provide superior prognostic utility for identifying CTO patients at higher risk of impaired CCC. These findings support the potential of SII as a valuable tool in clinical risk stratification and personalized treatment planning in coronary artery disease management.

### The role of FAR in coronary collateral circulation development

This study also identified the FAR as a significant and independent predictor of poorly-developed CCC in patients with CTO. As a composite biomarker derived from two key plasma proteins—FIB and ALB—FAR reflects both systemic inflammatory activity and nutritional status. It has recently emerged as a novel indicator in cardiovascular research. Elevated FIB and reduced ALB concentrations have each been independently associated with unfavorable cardiovascular outcomes, and their ratio has been linked to the extent and severity of coronary artery disease (32).

In the present cohort, FAR levels were significantly higher in patients with insufficient collateral vessel development. Multivariate logistic regression analysis confirmed FAR as an independent risk factor for impaired CCC. Furthermore, ROC curve analysis demonstrated that FAR had comparable diagnostic efficacy to that of the SII, supporting its utility as a moderate yet clinically relevant predictor. These findings suggest that an elevated FAR, indicative of a pro-inflammatory and hypo-nutritional state, may unfavorably influence the biological processes required for effective collateral formation.

The underlying mechanisms linking FAR to CCC impairment are likely multifactorial. FIB, a classical acute-phase reactant, plays a central role in vascular inflammation and hemostasis. It facilitates leukocyte-endothelial interactions, promotes platelet aggregation, and contributes to increased plasma viscosity, thereby exacerbating microvascular dysfunction and endothelial injury (33). FIB and its degradation products may infiltrate the subendothelial space, enhancing vascular permeability and lipid deposition, processes that accelerate atherogenesis. Additionally, FIB is implicated in the migration and proliferation of vascular smooth muscle and endothelial cells, which can disrupt vascular architecture and impair remodeling capacity.

In contrast, ALB exhibits anti-inflammatory, antioxidative, and antithrombotic functions that confer vascular protection (34). Hypoalbuminemia has been associated with increased cardiovascular morbidity and mortality, serving as a marker of both malnutrition and chronic inflammation. ALB stabilizes endothelial integrity, regulates plasma oncotic pressure, inhibits platelet aggregation, and mitigates oxidative stress. Reduced ALB levels thus contribute to increased blood viscosity, endothelial dysfunction, and pro-inflammatory conditions, all of which impair vascular homeostasis and neovascularization.

As a combined marker, FAR integrates these opposing physiological pathways. An elevated FAR likely signifies an internal environment characterized by excessive coagulation activity, endothelial damage, and limited angiogenic potential. Although prior studies have primarily explored the role of FAR in acute coronary syndromes (33), evidence regarding its association with chronic coronary pathology remains sparse. Our findings extend this understanding by demonstrating that FAR is negatively correlated with Rentrop grade, indicating its inverse association with the degree of collateral vessel development in CTO.

Emerging data underscore the importance of endothelial cell integrity and vascular remodeling in neovascularization and collateral growth (35). High FIB levels may compromise endothelial barrier function and promote thrombotic events, while reduced ALB concentrations may aggravate oxidative damage and facilitate low-density lipoprotein modification (36). FAR, by capturing both inflammatory and nutritional imbalances, offers a comprehensive snapshot of the vascular microenvironment. Its elevation may thus reflect a pathophysiological state unfavorable to collateral vessel formation.

In conclusion, FAR appears to be a reliable, easily obtainable, and clinically informative biomarker that encompasses both systemic inflammation and nutritional status. Its strong association with diminished CCC suggests that FAR may serve as a valuable tool for prognostic evaluation and risk stratification in CTO patients. Further prospective studies are needed to determine whether interventions targeting systemic inflammation or improving nutritional status can enhance collateral vessel development and optimize patient outcomes.

### Clinical implications

The results of this study highlight the potential clinical utility of both the SII and the FAR as practical, cost-effective biomarkers for the early detection of inadequate coronary collateral development in patients with CTO. As these markers are routinely obtainable through standard laboratory tests, their incorporation into everyday clinical practice is feasible. Elevated SII and FAR values may serve as early indicators of impaired collateral formation, thereby assisting clinicians in identifying high-risk individuals who may benefit from intensified surveillance or personalized therapeutic approaches. Moreover, integrating these indices into preprocedural assessments could enhance risk stratification and support decision-making regarding the need for more aggressive pharmacologic management or consideration of alternative revascularization strategies.

## Limitations

Despite its novel contributions, this study is subject to several limitations. First, it employed a retrospective, single-center design, which may introduce selection bias and limit the generalizability of the findings to broader populations. The study only included patients undergoing elective coronary angiography, which may not represent all patients with CTO. Second, inflammatory and nutritional biomarkers were measured only once at the time of admission, without serial evaluation, which restricts the ability to capture temporal changes in systemic inflammation or nutritional status. Repeated measurements over time could provide a more comprehensive assessment of these biomarkers and their dynamic changes. Third, while the reported AUC values are statistically significant, they indicate only moderate discriminatory performance, which may limit the clinical applicability of these biomarkers. Further research with larger, more diverse populations is needed to better assess their potential clinical relevance. Additionally, the assessment of CCC was based on the Rentrop classification, which, while commonly utilized, is a qualitative angiographic measure and may not accurately represent the functional efficacy of collateral vessels. Lastly, the study did not include follow-up data on long-term clinical outcomes, such as major adverse cardiovascular events or mortality, thus limiting conclusions regarding the prognostic implications of SII and FAR.

## Conclusion

In summary, this study demonstrates that elevated levels of SII and FAR are independently associated with poorly-developed CCC in patients with CTO. As indicators of systemic inflammation and metabolic balance, both markers may serve as accessible and clinically relevant tools for stratifying patient risk. Importantly, their combined use enhances predictive accuracy and may inform individualized treatment planning. Further prospective, multicenter studies with larger sample sizes and extended follow-up are warranted to validate these findings and to elucidate the long-term prognostic value of SII and FAR in the context of coronary artery disease.

## Data Availability

The raw data supporting the conclusions of this article will be made available by the authors, without undue reservation.
