# Supplementary material for: Systemic immune-inflammation index and fibrinogen-to-albumin ratio as predictors of coronary collateral circulation in chronic total occlusion patients
Source: Front Cardiovasc Med. 2026 Mar 31;13:1777321. doi: 10.3389/fcvm.2026.1777321 (PMC13076287; doi:10.3389/fcvm.2026.1777321)
Supplement: Supplementary file 1 [file Table1.docx]

Table 1 Baseline clinical characteristics and laboratory biomarkers between poorly-developed CCC group and well-developed CCC group

|  | ALL (n= 469) | Poorly-developed CCC group (n= 183) | Well-developed CCC group (n= 286) | P value |
| --- | --- | --- | --- | --- |
| Age, years | 68.50±3.78 | 68.59±3.60 | 68.44±3.90 | 0.677 |
| Male, n% | 270 (57.6%) | 103 (56.3%) | 167 (58.4%) | 0.722 |
| Smoking history, n% | 224 (47.8%) | 95 (51.9%) | 129 (45.1%) | 0.178 |
| Hypertension, n% | 235 (50.1%) | 98 (53.6%) | 137 (47.9%) | 0.271 |
| Diabetes mellitus, n% | 125 (26.7%) | 54 (29.5%) | 71 (24.8%) | 0.311 |
| Prior history of stroke, n% | 99 (21.1%) | 33 (18.0%) | 66 (23.1%) | 0.234 |
| SBP, mmHg | 134.68±18.88 | 135.49±17.81 | 134.15±19.51 | 0.456 |
| DBP, mmHg | 81.98±11.98 | 82.32±10.67 | 81.77±12.75 | 0.629 |
| Heart rate, bpm | 75.59±13.68 | 76.67±13.13 | 74.90±13.99 | 0.173 |
| Body mass index, kg/m^2^ | 25.07±2.67 | 24.99±2.26 | 25.12±2.90 | 0.611 |
| Laboratory biomarkers | | | | |
| White blood cell, ×10^9/L | 6.87±1.53 | 7.09±1.53 | 6.73±1.52 | 0.011 |
| Neutrophil count, ×10^9/L | 4.38±1.27 | 4.95±1.30 | 4.02±1.11 | <0.001 |
| lymphocyte count, ×10^9/L | 1.83±0.63 | 1.62±0.55 | 1.97±0.63 | <0.001 |
| Monocyte count, ×10^9/L | 0.51(0.45,0.57) | 0.50(0.45,0.56) | 0.52(0.45,0.58) | 0.203 |
| Eosinophil count, ×10^9/L | 0.09(0.06,0.12) | 0.09(0.06,0.13) | 0.09(0.06,0.12) | 0.346 |
| Basophil count, ×10^9/L | 0.03(0.02,0.04) | 0.03(0.02,0.04) | 0.03(0.02,0.04) | 0.681 |
| Red blood cell count, ×10^12/L | 4.68(4.49,4.88) | 4.71(4.50,4.89) | 4.67(4.46,4.88) | 0.285 |
| Hemoglobin concentration, g/L | 143.97(138.48,149.63) | 144.16(138.81,149.37) | 143.71(138.43,149.82) | 0.532 |
| Platelet count, ×10^9/L | 229.38±48.44 | 240.43±48.03 | 222.31±47.37 | <0.001 |
| Fasting blood glucose, mmol/L | 5.94(5.44,6.47) | 5.86(5.54,6.33) | 6.01(5.39,6.62) | 0.145 |
| Glycated hemoglobin, % | 6.10(5.63,6.56) | 6.06(5.69,6.45) | 6.12(5.59,6.68) | 0.193 |
| Fibrinogen, g/L | 3.01(2.83,3.19) | 3.19(3.02,3.36) | 2.90(2.71,3.07) | <0.001 |
| hs-CRP, mg/L | 3.81±2.01 | 5.17±2.18 | 2.93±1.29 | <0.001 |
| Blood urea nitrogen, mmol/L | 6.06(5.38,6.80) | 6.13(5.41,6.82) | 6.02(5.36,6.76) | 0.249 |
| Uric acid, μmol/L | 346.14(312.19,393.79) | 342.97(295.69,394.50) | 346.95(316.36,391.09) | 0.069 |
| Creatinine, μmol/L | 77.25(70.63,84.09) | 77.04(70.32,87.50) | 77.41(71.17,82.79) | 0.299 |
| eGFR, mL/min/1.73m^2 | 91.79(86.37,97.10) | 92.25(87.19,97.54) | 91.49(85.81,96.62) | 0.070 |
| ALT, U/L | 21.60(16.91,26.58) | 22.29(17.51,26.89) | 21.33(16.11,25.91) | 0.066 |
| AST, U/L | 19.03(16.28,22.16) | 19.03(16.82,21.62) | 19.04(15.47,22.77) | 0.370 |
| Albumin, g/L | 41.28(40.09,42.58) | 41.03(39.86,42.61) | 41.47(40.18,42.56) | 0.079 |
| Globulin, g/L | 26.14(25.07,27.14) | 26.16(24.85,27.05) | 26.13(25.19,27.17) | 0.266 |
| Total bilirubin, μmol/L | 12.14(10.61,13.91) | 12.33(10.72,14.13) | 11.98(10.58,13.72) | 0.145 |
| Direct bilirubin, μmol/L | 3.34(2.89,3.87) | 3.37(2.83,3.85) | 3.34(2.91,3.87) | 0.735 |
| Indirect bilirubin, μmol/L | 8.61(7.49,9.78) | 8.37(7.28,9.77) | 8.81(7.60,9.77) | 0.186 |
| Triglycerides, mmol/L | 1.58(1.33,1.86) | 1.60(1.34,1.93) | 1.56(1.32,1.84) | 0.162 |
| Total cholesterol, mmol/L | 3.77(3.52,4.06) | 3.84(3.46,4.18) | 3.74(3.55,4.02) | 0.103 |
| HDL-C, mmol/L | 0.855(0.795,0.908) | 0.903(0.842,0.959) | 0.822(0.763,0.883) | <0.001 |
| LDL-C, mmol/L | 2.483(2.207,2.775) | 2.472(2.227,2.818) | 2.488(2.201,2.759) | 0.401 |
| Apolipoprotein B, mg/dL | 84.555(76.679,91.374) | 84.908(76.291,93.095) | 84.189(76.791,90.700) | 0.380 |
| Lipoprotein(a), mg/L | 193.840(97.842,288.977) | 202.178(100.181,310.371) | 191.267(97.876,276.421) | 0.307 |
| cTNI, ng/ml | 0.010(0.005,0.016) | 0.010(0.005,0.016) | 0.010(0.005,0.016) | 0.734 |
| NT-ProBNP, pg/ml | 482.99±715.69 | 508.04±762.34 | 466.97±683.70 | 0.545 |
| SII, 10^9/L | 515.3 (359.6, 682.7) | 670.7 (360.2, 986.8) | 473.4 (358.6, 583.8) | <0.001 |
| FAR | 0.075 (0.069, 0.081) | 0.082 (0.079, 0.085) | 0.071 (0.067, 0.074) | <0.001 |
| NLR | 2.58±1.19 | 3.15±1.52 | 2.22±0.71 | <0.001 |
| PLR | 157.33±19.63 | 154.45±16.42 | 159.17±21.23 | 0.011 |
| LVEF, % | 50.61±5.10 | 49.92±4.74 | 51.06±5.27 | 0.018 |

Continuous variables were expressed as mean±SD, or median (interquartile range). Categorical variables were expressed as number (percentage).

Abbreviations: CCC, coronary collateral circulation; SBP, systolic blood pressure; DBP, diastolic blood pressure; hs-CRP, high-sensitivity C-reactive protein; eGFR, estimated glomerular filtration rate; ALT, alanine aminotransferase; AST, aspartate aminotransferase; HDL-C, high-density lipoprotein cholesterol; LDL-C, low-density lipoprotein cholesterol; cTNI, cardiac troponin I; NT-ProBNP, N-terminal pro-B-type natriuretic peptide; SII, systemic immune-inflammation index; FAR, fibrinogen-to-albumin ratio; NLR, neutrophil-to-lymphocyte ratio; LVEF, left ventricular ejection eraction; DAPT, dual antiplatelet therapy; ACEI, angiotensin-converting enzyme inhibitor; ARB, angiotensin-receptor blocker; PLR, platelet-to-lymphocyte ratio;

Table 2 Angiographic findings and Pre-admission medication

|  | ALL (n= 469) | Poorly-developed CCC group (n= 183) | Well-developed CCC group (n= 286) | P value |
| --- | --- | --- | --- | --- |
| Angiographic findings | | | | |
| CTO lesion location, n% | | | | |
| LAD | 207 (44.1%) | 90 (49.2%) | 117 (40.9%) | 0.096 |
| LCX | 130 (27.7%) | 49 (26.8%) | 81 (28.3%) | 0.795 |
| RCA | 242 (51.6%) | 82 (44.8%) | 160 (55.9%) | 0.023 |
| Multi-vessel CTO lesions, n% | 63 (13.4%) | 22 (12.0%) | 41 (14.3%) | 0.563 |
| Gensini score | 62.00(47.00,78.00) | 42.00(34.00,51.00) | 75.00(65.00,86.00) | <0.001 |
| Pre-admission medication | | | | |
| DAPT, n% | 418 (89.1%) | 168 (91.8%) | 250 (87.4%) | 0.181 |
| Statins, n% | 347 (74.0%) | 139 (76.0%) | 208 (72.7%) | 0.503 |
| ACEI or ARB, n% | 220 (46.9%) | 78 (42.6%) | 142 (49.7%) | 0.163 |
| Beta-blockers, n% | 237 (50.5%) | 89 (48.6%) | 148 (51.7%) | 0.573 |
| Aldosterone antagonists, n% | 41 (8.7%) | 18 (9.8%) | 23 (8.0%) | 0.614 |
| Nitrates, n% | 205 (43.7%) | 88 (48.1%) | 117 (40.9%) | 0.151 |
| Calcium channel blockers, n% | 88 (18.8%) | 38 (20.8%) | 50 (17.5%) | 0.443 |

Continuous variables were expressed as median (interquartile range). Categorical variables were expressed as number (percentage).

Abbreviations: CCC, coronary collateral circulation; LAD, left anterior descending coronary artery; LCX, left circumflex coronary artery; RCA, right coronary artery; CTO, chronic total occlusion; ACEI, angiotensin-converting enzyme inhibitor; ARB, angiotensin-receptor blocker;

Table 3 Univariate and multivariate regression analyses for the influential factors of poorly-developed CCC formation in CTO patients

|  | Univariate Analysis | | | Multivariate Analysis | | | |
| --- | --- | --- | --- | --- | --- | --- | --- |
| Variables | OR | 95% CI | P value | OR | 95% CI | P value | VIF |
| White blood cell, ×10^9/L | 0.991 | 0.964-1.027 | 0.933 |  |  |  | 1.13 |
| Neutrophil count, ×10^9/L | 1.071 | 0.583-1.982 | 0.825 |  |  |  | 1.31 |
| lymphocyte count, ×10^9/L | 0.933 | 0.535-1.637 | 0.817 |  |  |  | 1.18 |
| Platelet count, ×10^9/L | 1.205 | 0.681-2.128 | 0.531 |  |  |  | 1.25 |
| Fibrinogen, g/L | 0.827 | 0.412-1.675 | 0.527 |  |  |  | 1.12 |
| hs-CRP, mg/L | 1.737 | 0.932-3.136 | 0.075 |  |  |  | 1.27 |
| HDL-C, mmol/L | 0.773 | 0.332-1.628 | 0.537 |  |  |  | 1.05 |
| SII, 10^9/L | 4.275 | 2.721-6.467 | ＜0.001 | 3.121 | 1.827-5.537 | ＜0.001 | 7.48 |
| FAR | 1.626 | 1.221-2.275 | 0.002 | 2.118 | 1.248-3.227 | 0.001 | 4.52 |
| NLR | 1.073 | 0.831-1.337 | 0.437 |  |  |  | 7.57 |
| PLR | 1.074 | 0.947-1.271 | 0.147 |  |  |  | 8.20 |
| LVEF, % | 0.937 | 0.901-1.036 | 0.337 |  |  |  | 1.37 |
| CTO lesion location at RCA | 4.026 | 2.247-7.745 | ＜0.001 | 2.471 | 0.974-4.327 | 0.073 | 3.22 |
| Gensini score | 1.014 | 1.004-1.047 | 0.025 | 1.000 | 0.943-1.044 | 0.936 | 2.54 |

Abbreviations: CCC, coronary collateral circulation; CTO, chronic total occlusion; OR, odds ratio; CI, confidence interval; RCA, right coronary artery; SII, systemic immune-inflammation index; FAR, fibrinogen-to-albumin ratio; NLR, neutrophil-to-lymphocyte ratio; LVEF, left ventricular ejection eraction; PLR, platelet-to-lymphocyte ratio; VIF, variance inflation factor;

Table 4 Comparison of SII and FAR across Rentrop grades

|  | Rentrop grade 0 (n= 84) | Rentrop grade 1 (n= 99) | Rentrop grade 2 (n= 161) | Rentrop grade 3 (n= 125) | P value |
| --- | --- | --- | --- | --- | --- |
| SII, 10^9/L | 647.86 (405.28, 1039.08) | 688.20 (344.55, 938.05) | 482.53 (374.67, 592.81) | 452.48 (341.28, 560.45) | 0.799, <0.001, 0.092 |
| FAR | 0.085 (0.084, 0.088) | 0.079 (0.077, 0.081) | 0.073 (0.071, 0.077) | 0.067 (0.064, 0.068) | <0.001 |

Continuous variables were expressed as median (interquartile range).

P values were calculated using independent samples t-test for SII and Mann–Whitney U test for FAR across sequential Rentrop grade comparisons (0 vs 1, 1 vs 2, 2 vs 3).

SII: Among the pairwise comparisons between Rentrop grades, only the difference between Grade 1 and Grade 2 was statistically significant (P < 0.001). Grade 0 vs 1 (P = 0.799) and Grade 2 vs 3 (P = 0.092) were not statistically significant.FAR: All sequential group comparisons are statistically significant (P < 0.001).

SII, systemic immune-inflammation index; FAR, fibrinogen-to-albumin ratio;

Table 5 ROC curve analysis of individual and combined indicators in predicting poorly-developed CCC formation in CTO patients

|  | AUC(95%CI) | Best cutoff | Sensitivity | Specificity | P value | | |
| --- | --- | --- | --- | --- | --- | --- | --- |
|  |  |  |  |  | SII | FAR | Combined predictor |
| SII | 0.67 (0.61–0.72) | 631.51 | 50.8% | 81.4% | - | 0.743 | 0.001 |
| FAR | 0.68 (0.63–0.73) | 0.077 | 53.4% | 73.2% | 0.743 | - | 0.004 |
| Combined predictor | 0.73 (0.68–0.78) | - | 59.8% | 75.7% | 0.001 | 0.004 | - |

CI, confidence interval; ROC, receiver operator characteristic; AUC, area under the curve;

SII, systemic immune-inflammation index; FAR, fibrinogen-to-albumin ratio;
