# Supplementary material for: Systemic immune-inflammation index and fibrinogen-to-albumin ratio as predictors of coronary collateral circulation in chronic total occlusion patients
Source: Front Cardiovasc Med. 2026 Mar 31;13:1777321. doi: 10.3389/fcvm.2026.1777321 (PMC13076287; doi:10.3389/fcvm.2026.1777321)
Supplement: Supplementary file 2 [file Table2.docx]

**Figure Legends**

**Figure 1.** Study flowchart.

Abbreviations: CCC, coronary collateral circulation; CTO, chronic total occlusion; CAG, coronary angiography;

**Figure 2**. Comparisons of SII and FAR Levels across Rentrop grades.

(A) Distribution of SII across Rentrop grade 0 to 3 groups. (B) Distribution of FAR across Rentrop grades. Data are presented as box plots with overlaid individual data points. Statistically significant differences (P < 0.001) are indicated above the brackets.

Abbreviations:SII, systemic immune-inflammation index; FAR, fibrinogen-to-albumin ratio;

**Figure 3.**  ROC curves for SII, FAR, and the combined predictor in predicting poorly-developed CCC formation in CTO patients.

Abbreviations: ROC, receiver operating characteristic; CCC, coronary collateral circulation; CTO, chronic total occlusion; SII, systemic immune-inflammation index; FAR, fibrinogen-to-albumin ratio;

**Figure. Central Illustration.** Prognostic Role of SII and FAR for CCC in CTO Patients.

This schematic summarizes the association between SII and FAR with the development of CCC in patients with CTO. Patients with poorly-developed CCC exhibited significantly higher levels of SII and FAR compared to those with well-developed CCC. SII and FAR were independent predictors of inadequate collateral formation, and their combination improved diagnostic accuracy (AUC = 0.73). These markers reflect systemic inflammation and nutritional status and may serve as practical tools for clinical risk stratification and therapeutic decision-making.

Abbreviations: SII, systemic immune-inflammation index; FAR, fibrinogen-to-albumin ratio; FIB, fibrinogen; ALB, albumin; CCC, coronary collateral circulation; CTO, chronic total occlusion.
