# Supplementary material for: Systemic immune-inflammation index and fibrinogen-to-albumin ratio as predictors of coronary collateral circulation in chronic total occlusion patients
Source: Front Cardiovasc Med. 2026 Mar 31;13:1777321. doi: 10.3389/fcvm.2026.1777321 (PMC13076287; doi:10.3389/fcvm.2026.1777321)
Supplement: Supplementary file 3 [file Table3.docx]

Dear Editor,

We are pleased to submit our manuscript entitled

“Prognostic Value of Systemic Immune-Inflammation Index and Fibrinogen-to-Albumin Ratio for Coronary Collateral Circulation in Patients with Chronic Total Occlusion”for consideration for publication in the Frontiers in Cardiovascular Medicine.

This retrospective, single-center observational study was conducted in 469 patients diagnosed with chronic total occlusion (CTO) of a major coronary artery. The objective was to investigate the relationship between two systemic biomarkers—systemic immune-inflammation index (SII) and fibrinogen-to-albumin ratio (FAR)—and the development of coronary collateral circulation (CCC), assessed by the Rentrop classification.

Our findings demonstrated that patients with poorly-developed CCC exhibited significantly elevated SII and FAR levels compared to those with well-developed CCC. Both markers remained independent predictors of impaired collateral formation after adjustment for potential confounders. Notably, the combined model of SII and FAR yielded improved diagnostic performance (area under the curve [AUC] = 0.73). These results suggest that SII and FAR may serve as accessible and cost-effective biomarkers for evaluating arteriogenic capacity and optimizing risk stratification in CTO patients.

To our knowledge, this is the first study to assess the combined prognostic value of SII and FAR in relation to CCC in the setting of CTO. These findings provide new insights into the inflammatory and nutritional mechanisms underlying coronary collateral formation and may contribute to more personalized management strategies in interventional cardiology.

This manuscript is original, has not been previously published, and is not under consideration elsewhere. All authors have read and approved the final version. There are no conflicts of interest to disclose. The study protocol was approved by the Ethics Committee of Xiangtan Central Hospital (Approval No. X2019523), and written or verbal informed consent was obtained from all participants in accordance with the Declaration of Helsinki (2013 revision).

We believe this manuscript will be of interest to your readership, particularly those engaged in cardiovascular research and clinical practice. We sincerely hope you will consider our submission for publication in your esteemed journal.

Thank you for your time and consideration.

Sincerely,

Mingxing Wu, MD

On behalf of all co-authors

Department of Cardiology

Xiangtan Central Hospital, Hunan, China

Email: [heartwl@126.com](mailto:heartwl@126.com)
